# Serine and one-carbon metabolism sustain non-melanoma skin cancer progression

**DOI:** 10.1038/s41420-023-01398-x

**Published:** 2023-03-24

**Authors:** Angela Cappello, Alessandro Zuccotti, Mara Mancini, Giulia Tosetti, Luca Fania, Francesco Ricci, Gerry Melino, Eleonora Candi

**Affiliations:** 1 Department of Experimental Medicine, University of Rome “Tor Vergata”, 00133 Rome, Italy; 2 Istituto Dermopatico dell’Immacolata, IDI-IRCCS, 00167 Rome, Italy

**Keywords:** Cancer metabolism, Skin cancer

## Abstract

Non-melanoma skin cancer (NMSC) is a tumor that arises from human keratinocytes, showing abnormal control of cell proliferation and aberrant stratification. Cutaneous basal cell carcinoma (cBCC) and cutaneous squamous cell carcinoma (cSCC) are the most common sub-types of NMSC. From a molecular point of view, we are still far from fully understanding the molecular mechanisms behind the onset and progression of NMSC and to unravel targetable vulnerabilities to leverage for their treatment, which is still essentially based on surgery. Under this assumption, it is still not elucidated how the central cellular metabolism, a potential therapeutical target, is involved in NMSC progression. Therefore, our work is based on the characterization of the serine anabolism/catabolism and/or one-carbon metabolism (OCM) role in NMSC pathogenesis. Expression and protein analysis of normal skin and NMSC samples show the alteration of the expression of two enzymes involved in the serine metabolism and OCM, the Serine Hydroxy-Methyl Transferase 2 (SHMT2) and Methylen-ThetraHydroFolate dehydrogenase/cyclohydrolase 2 (MTHFD2). Tissues analysis shows that these two enzymes are mainly expressed in the proliferative areas of cBCC and in the poorly differentiated areas of cSCC, suggesting their role in tumor proliferation maintenance. Moreover, in vitro silencing of SHMT2 and MTHFD2 impairs the proliferation of epidermoid cancer cell line. Taken together these data allow us to link the central cellular metabolism (serine and/or OCM) and NMSC proliferation and progression, offering the opportunity to modulate pharmacologically the involved enzymes activity against this type of human cancer.

## Introduction

Non-melanoma skin cancer (NMSC) represents the most common diagnosed cancer worldwide [1]. Cutaneous basal cell carcinoma (cBCC) and cutaneous squamous cell carcinoma (cSCC) are the most representative types of non-melanoma skin cancer. In general, cBCC and cSCC present different molecular pathogenesis, growth, and aggressiveness. Although there is a high incidence of these tumors [2–4], the survival is quite high because they are typically recognized in time, but the absolute number of deaths is similar to that of melanoma [5].

The cBCC is characterized by cancer cells reminiscent of the proliferative basal cells of the epidermis; preferentially arises from interfollicular stem cells, hair follicle infundibulum cells or hair follicle bulge cells, and it is the less aggressive NMSC [6, 7]; usually, cBCC shows a limited capability to invade and destroy the adjacent tissues and for these reasons it shows a low degree of malignancy [8]. Several risk factors related to cBCC insurgence have been identified, i.e. immunosuppression, genetic syndromes and skin photosensitivity (Fitzpatrick skin types I and II); but ultraviolet radiation (UV) represents the most prominent and primary risk factor for cBCC development, although the link between cBCC and UV exposure is still quite controversial [1]. Different studies demonstrate that a cumulative UV exposure determines changes in skin protein expression [9]. In fact, a chronic UV exposure leads to the increase of Reactive Oxygen Species (ROS) production, DNA damage (pyrimidine dimers) and p53-dependent apoptosis in human normal keratinocytes [10]. These molecular changes are often followed by tissue alterations, such as hyperproliferation and epidermal hyperplasia, representing the initial steps of NMSC development, which have also been proven to be fundamental in animal models of the disease [11, 12].

The cSCC develops from abnormal epidermis squamous cells and it is characterized by a strong migration and invasion capability. Tumor aggressiveness and recurrence depends on histological features such as its pathological grade and on anatomic localization. Patients with this type of cancer have a ten-year survival of about 90%, but the recurrences are very frequent. Therefore, cSCC represents the second cause of death for skin cancer after melanoma [13]. Among the risk factors for cSCC development we can mention skin photosensitivity (Fitzpatrick skin types I and II), immunosuppression, HIV, Human Papilloma Virus (HPV) infection, a chronic UV exposure and a previous story of actinic keratosis (AK) [14–17]. As well as cBCC, the chronic UV exposure is the most important environmental factor involved in cSCC insurgence; indeed, this tumor often arises on sun-exposed area [18]. In addition, different studies indicate that AK, typically manifested on skins chronically exposed to the sun-light, is a premalignant lesion that often herald cSCC development [19]. For this reason, AK is considered the most significant predictive factor for cSCC development, beneath not already identified prognostic signatures have been defined at a molecular level [20, 21]. Surgical excision is the first therapy against NMSC; however, some alternative approaches can be used, i.e. photodynamic therapy, cryotherapy, topical imiquimod 5%, and topical diclofenac sodium 3% [8, 22, 23]. SCCs also arises from squamous cells that line in the mucosal surfaces of the head and neck (HNSCC) including oral cavity, pharynx, larynx, tongue as well an in lung [24, 25]. HNSCCs are the sixth most common cancer worldwide [26]. Tobacco and alcohol are the primary risk factors, as well as virus infection [27–30]. Similarly to cSCCs, HNSCC incidence is continuously rising, and it is expected to increase by 30% by 2030. Unlike cSCCs, one of the main factors affecting the low survival rate of HNSCC, is the high percentage of diagnosis at advanced stage, being the recurrence rate of about 50% during the first 2 years from diagnosis. For this reason, several clinical and/or pathological parameters have been indicated to predict prognosis, recurrence, and survival, and to drive the therapeutic choice [31–37]. Notably, cSCCs and HNSCCs display hallmarks of solid tumors mildly responsive to systemic immunotherapy [38–45]. Thus, immunotherapy with PD-1 blockade has emerged as the latest standard-of-care treatment strategy developed for advanced and metastatic SCCs. A crucial step of NMSC and HNSCC development is the alteration of normal epidermal cell proliferation; indeed, several studies report that cancer cells can adapt their metabolism to achieve this effect [46–52]. The term one-carbon metabolism (OCM) refers to a complex metabolic pathway involved in the generation of one-carbon units (hydroxymethyl groups) used by the cells for the biosynthesis of fundamental anabolic precursors, for cellular redox homeostasis and for methylation reactions [53]. The importance of OCM in cancer was recognized when it was observed that a diet poor of folate in children with acute leukaemia was able to reduce the number of their leukaemic cells but, at date, the involvement of this metabolism in the pathogenesis of different cancer types is still under evaluation [54]. In general, the alteration of serine and OCM is strongly associated with different types of cancer with a high rate of cellular proliferation [55]. The OCM reactions have a pivotal role in the production of pyrimidine and purine nucleotides (DNA and RNA synthesis), S-adenosylmethionine (SAM), necessary for DNA methylation and to produce reduced GSH for ROS scavenging [56]. Closely related to OCM is the serine biosynthesis pathway, an alternative route of glycolysis in which glucose is converted in serine through the action of three enzymes: phosphoglycerate dehydrogenase (PHGDH), phosphoserine aminotransferase 1 (PSAT1) and phosphoserine phosphatase (PSPH) [57]. After the generation of serine form glucose, the amino acid is metabolized both in the cytosol and in the mitochondria through the activity of Serine Hydroxy-Methyl Transferase enzymes (SHMT1 and SHMT2 enzymes, respectively), thus representing the major source of one-carbon units. In general, the interconnection of serine anabolic/catabolic reactions and OCM are a crucial metabolic hub for cellular energy production, regulation of proliferation rate, and cellular differentiation and for these reasons it is essential to describe a possible way to reprogram these metabolisms in cancer. This work lays the foundation for identifying the link between serine and/or OCM metabolism and abnormal proliferation of NMSC, of the most aggressive (cSCC), trying to identify possible metabolic vulnerabilities against this type of cancer.

## Results

### Serine and OCM enzymes are differentially expressed in NMSC

**Fig. 1:**
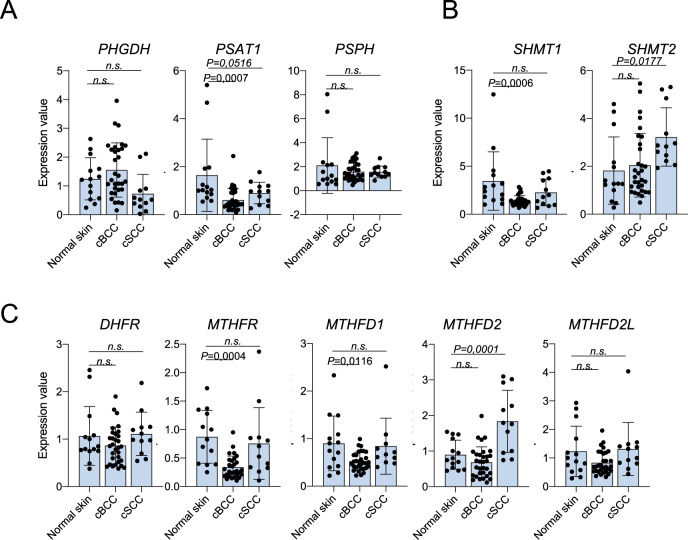
Expression analysis of serine de novo biosynthesis, serine anabolism/catabolism and one-carbon metabolism enzymes in human samples of normal skin, cBCC and cSCC. **A** Expression analysis through RTqPCR of serine biosynthesis, **B** *SHMT1* and *SHMT2* and **C** one-carbon metabolism enzymes in a cohort of human samples collected from IDI-IRCCS -normal skin (*n* = 14), cBCC (*n* = 31), cSCC (*n* = 12). The *p*-value was obtained using Student’s *t* test. Values were considered significant when the *p*-value < 0.05, n.s. not significant.

Although the involvement of serine metabolism and OCM in the proliferation and progression of different human malignancies [46, 57] is, at date, quite well described, its pathogenic role in NMSC has yet to be investigated. We analyzed the expression of the enzymes involved in serine *de novo* biosynthesis (PHGDH, PSAT1 and PSPH), the enzymes involved in serine anabolism/catabolism reactions in the cytoplasm and in the mitochondria (SHMT1 and SHMT2, respectively) and the cytoplasmic and mitochondrial enzymes involved in the formation of the cofactor tetrahydrofolate (THF), DHFR, MTHFR, MTHFD1, MTHFD2, MTHFD2L. The mRNA expression was analyzed using collected human samples of normal skin (*n* = 14), cBCC (*n* = 31) and cSCC (*n* = 12), Fig. 1. mRNA levels, evaluated through RT-qPCR (Fig. 1A), showed the mitochondrial enzymes *SHMT2* and *MTHFD2* differentially expressed in tumor samples of cSCC, the most aggressive NMSC. While in cBCC we observed the reduced expression of *PSAT1*, *SHMT1*, *MTHFR*, *MTHFD1*. To expand these results, we interrogated a publicly available dataset (GSE7553) of cBCC (*n* = 15), cSCC (*n* = 11) and normal skin (n = 4) as the healthy control. The mRNA expression values are represented by boxplots in Supplementary Fig. S1A–C. The small number of samples did not allow us to find striking changes in NMSC gene expression for many of the enzymes analyzed, compared to their expression in normal skin, except for two genes whose expression is significantly reduced in cBCC (*SHMT1 P* = 0,046 and *MTHFD2L P* = 0,0004). Only one gene expression was increased in cSCC (*PSPH P* = 0,0295) compared to normal skin. These data indicate a possible involvement of serine and OCM metabolism in NMSC.

### SHMT2 and MTHFD2 are differentially expressed in cSCC and BCC

**Fig. 2:**
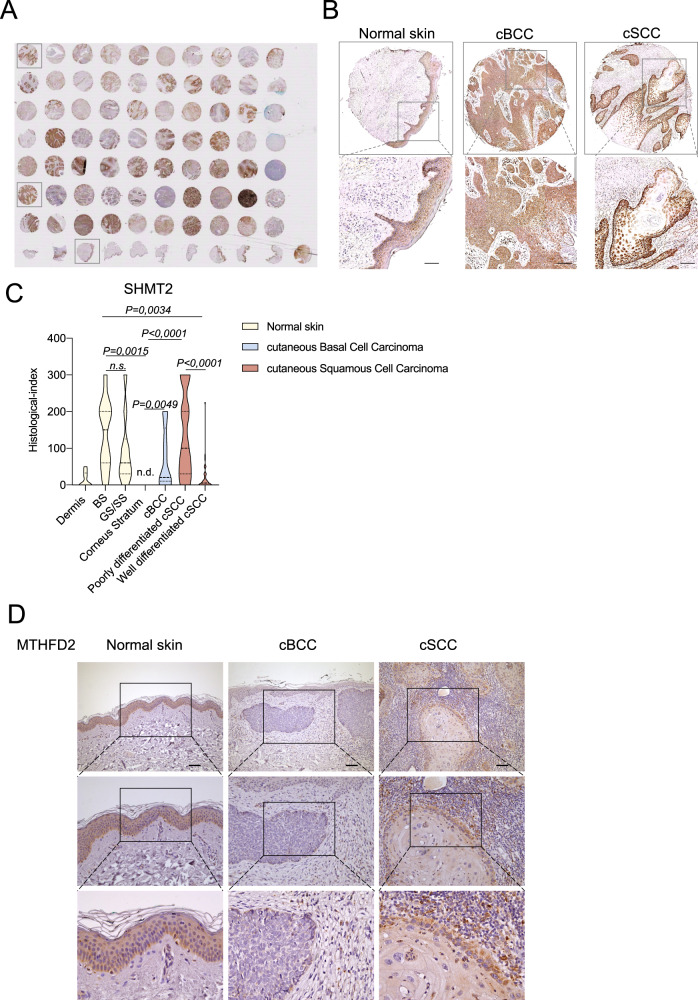
SHMT2 and MTHFD2 are differentially expressed in normal skin and Non-Melanoma skin cancer. **A** Immunohistochemical staining for SHMT2 performed on Tissue Micro Array (TMA), containing cases of normal skin (*n* = 10), cutaneuos Basal Cell Carcinoma (*n* = 14) and cutaneous Squamous Cell carcinoma (*n* = 43). **B** SHMT2 expression in 3 representative samples of normal skin, cBCC and cSCC. **C** Histological index of SHMT2 expression in normal skin (divided into Dermis, Basal Stratum-BS-, Granulosum Stratum-GS-, Spinous Stratum-SS-, and Corneum Stratum), cBCC and cSCC (divided into Well differentiated cSCC and Poorly differentiated cSCC). **D** Immunohistochemical staining for MTHFD2 performed on FFPE tissues of normal skin, cutaneuos Basal Cell Carcinoma (cBCC) and cutaneous Squamous Cell carcinoma (cSCC). Scale Bar = 100 μm. The *p*-value was obtained using Student’s *t* test. Values were considered significant when the *p*-value < 0.05, n.s. not significant.

To further investigate the role of the modulated enzymes, additional NMSC samples from commercially available Tissue Micro Array (TMA), were analyzed (Fig. 2A). Specifically, 14 and 43 samples of cBCC and cSCC were analyzed for SHMT2 expression, respectively. NMSC samples were compered to 10 samples of normal skin. The immunohistochemistry performed on TMA, indicates an increased expression of SHMT2 in the basal layer of the skin compared to the upper differentiated layers. In cBCC representative samples SHMT2 was detected in all tumor cells, in contrast to cSCC representative samples where the expression of SHMT2 decreased in the differentiated area of the tumor (Fig. 2B). This evidence was confirmed by the analysis of the histological score, obtained by a combination of the SHMT2 intensity expression and the percentage of the tumor sample positive for its expression. The differentiated upper layers of human skin have a reduced SHMT2 expression compared to the basal undifferentiated layer and similarly SHMT2 is significantly less expressed in the well differentiated areas of cSCC compared to the poorly differentiated ones (Fig. 2C). Globally, SHMT2 expression in cBCC is strongly up regulated compared to the last differentiated layer of human skin, the corneum stratum.

MTHFD2 staining shows that the enzyme is abundantly expressed in the basal and suprabasal levels of human normal skin and in poorly differentiated areas of cSCC, while it is less expressed in BCC (Fig. 2D). These data are consistent to the MTHFD2 expression at mRNA levels (Fig. 1). These observations suggest that serine catabolism/anabolism and OCM enzymes are differently involved in cBCC and cSCC.

### In vitro modulation of the mitochondrial enzymes SHMT2 and MTHFD2, acts on cSCC proliferation

**Fig. 3:**
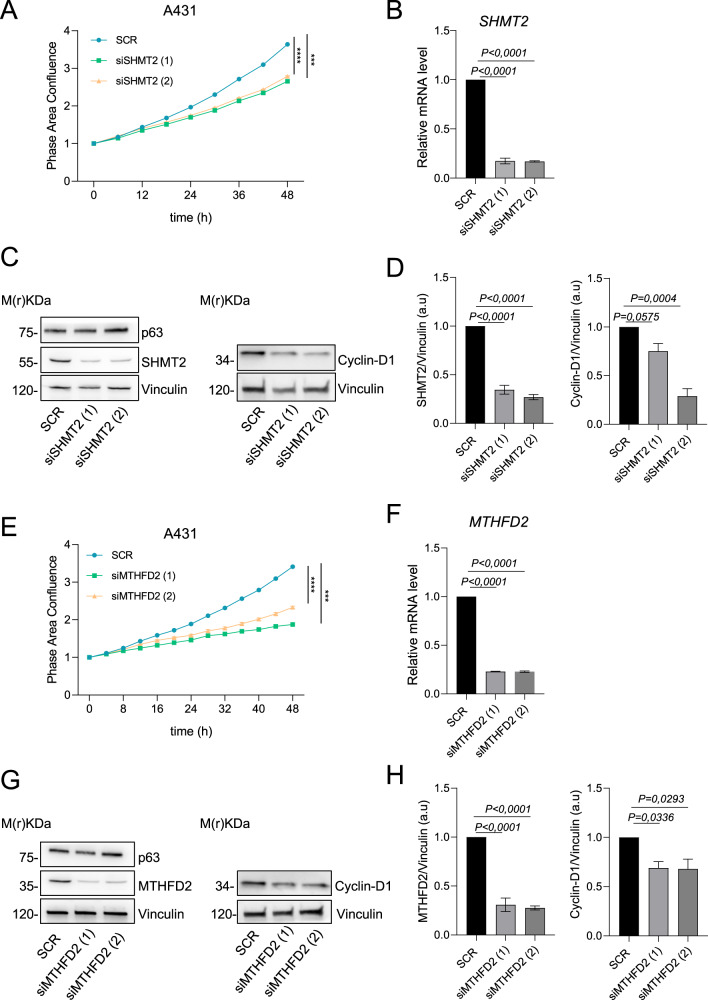
SHMT2 and MTHFD2 silencing effects on A431 proliferation. **A** Confluence analysis of A431 cells after *SHMT2* silencing. **B** *SHMT2* expression analysis through qPCR after silencing. **C** Western blot analysis after SHMT2 silencing, one representative experiment of 3 is shown. **D** Relative ratio of SHMT2 and Cyclin-D1 expression normalized to that of Vinculin. **E** Confluence analysis of A431 cells after *MTHFD2* silencing. **F** *MTHFD2* expression analysis through qPCR after silencing. **G** Western blot analysis after MTHFD2 silencing, one representative experiment of 3 is shown in **H**. Relative ratio of MTHFD2 and Cyclin-D1 expression normalized to that of Vinculin. The *p*-value was obtained using Student’s *t* test and One-Way Anova. Values were considered significant when the *p*-value < 0.05, n.s. not significant.

Next, we focused the attention on the SHMT2 and MTHFD2, the two mitochondrial enzymes significantly modulated at mRNA and protein level in cSCCs. The mitochondrial enzymes SHMT2 and MTHFD2 are involved in fundamental metabolic pathways, serine and THF metabolism, respectively. To characterize their functional role in cancer, we decided to modulate their expression in A431 cells, using two transient and specific siRNAs. The evaluation of cellular proliferation was made using Incucyte® Live-Cell analysis. The confluence curves show a significant reduction of A431 growth after in vitro silencing of *SHMT2* and *MTHFD2* and confirms the possible involvement of these enzymes in controlling cSCC proliferation (Fig. 3A–H). As control, by RT-qPCR and western blot, we confirmed SHMT2 and MTHFD2 knockdown (Fig. 3B–F, C–G). In line with the growth curve results, western blots confirmed the reduction in the expression of cyclin D1, after 72 hours silencing of *SHMT2* and *MTHFD2* (Fig. 3C, D, G, H). These data globally confirm the involvement of serine metabolism and OCM in NMSC proliferation, serving as hypothetical targets against this tumor.

## Discussion

Human epidermis is a multilayer stratified epithelium, consisting of keratinocytes and melanocytes, which represent the most common cell types of the tissue. In particular, the physiological keratinocyte proliferation, differentiation and stratification are essential for normal epidermis formation and homeostasis [58]. Alterations of this phenomenon and the insurgence of others such as the epithelial-to-mesenchymal transition are responsible of different skin disorders, including NMSC [59]. A strict metabolic regulation is crucial for normal cell fate and our work focus on the importance of this metabolic control in NMSC. Serine and OCM represent the major source of anabolic precursors for cellular energy production, DNA methylation and redox balance [60–62]. SHMT2 and MTHFD2 enzymes are two essential turning points of serine and OCM, respectively. SHMT2 catalyzes, in the mitochondrial compartment, the reversible conversion of serine and THF to glycine and 5,10-methylen-THF to guarantee the one-carbon units for the THF cycle [63], while the mitochondrial enzyme MHFD2 is responsible of the 10-formylTHF production through two different reactions catalyzed by the dehydrogenase and cyclohydrolase domains [64].

The enzymatic activity of SHMT2 and MTHFD2 is often deregulated in different types of human cancer affecting the cellular mechanism above-mentioned (DNA methylation and synthesis, redox balance, and protein synthesis) [65, 66]. Recent studies have led to the development of chemical inhibitors specifically targeting SHMT2 and MTHFD2, potentially promising as treatments to impair proliferation and aggressiveness of cancer cells [67]. To date, nothing is known about the involvement of these enzymes in NMSC progression; our preliminary bioinformatic analysis showed a trend to the de-regulation of the genes involved in serine and OCM in NMSC, if compared to normal skin (Supplementary Fig. 1A–C). However, RT-qPCR analysis on a larger number of samples increased the level of this evidence since *SHMT2* and *MTHFD2* are significantly up-regulated in cSCC, the more aggressive NMSC (Fig. 1A, C). The analysis of additional samples of NMSC on TMA confirms that SHMT2 is more expressed in the proliferative layer of human skin compared to the differentiated ones (Fig. 2B, C). Interestingly, SHMT2 is expressed in all cBCC cells, in contrast to cSCC, where SHMT2 is significantly downregulated in the more differentiated areas of the tumor (Fig. 2C). In addition, similarly to SHMT2, also MTHFD2 is more expressed in proliferating cells of cSCC (Fig. 2D), suggesting that these cells rely on this metabolism to sustain their growth. Finally, the in vitro modulation of SHMT2 and MTHFD2 determines the reduction of A431 cells ability to undergo to a complete cellular confluence (Fig. 3A, E) and the decrease of the proliferation markers cyclin D1 (Fig. 3C, D, G, H). Taken together these data show the fundamental role of serine and OCM, in particular of the mitochondrial enzymes SHMT2 and MTHFD2, in NMSC pathogenesis suggesting hypothetical metabolic vulnerabilities on which to act in the treatment of these types of tumors.

## Material and methods

### Cell culture, transfection, and proliferation assay

A431 cells was cultured in DMEM (Corning, Cat. No 10-102-CVR). For the Phase Area Confluence analysis, Incucyte® Live-Cell Analysis (Essen BioScience) was used. For siRNA-mediated knockdown experiments, 2,5 × 10^5^ cells were seeded in 60 mm plates and transfected with specific siRNAs (Supplementary Table 2) using Lipofectamine RNAiMAX transfection reagent (Invitrogen). The cells were collected 72 h after transfection.

### Immunohistochemical staining and histological scoring

TMA sections (purchased from US Biomax, Cat. No. SK801c, Rockville, MD, USA) were dewaxed for 2 h at 60 °C, then treated with Bio-Clear (Bio Optica) and rehydrated with alcohol scale and ddH_2_O. Immunohistochemical staining was performed using UltraTek HRP anti-polyvalent (DAB) (ScyTek laboratories, Cat. No. AMF080). Samples were boiled at 95 °C for 15 min in sodium citrate buffer at pH 6.0 for antigen retrieval. Anti-SHMT2 antibody was incubated (1:100, Sigma Cat. No. HPA020549) for 1 h. Anti-MTHFD2 antibody was incubated (1:500, Abcam Cat. No. ab151447) for 1 h. Sections were counterstained with Mayer’s haematoxylin (Bioptica, Cat. No. 05-06002E).

Histological index was visually analyzed in a blinded manner by a pathologist using a semi-quantitative method. NMSC cases were analyzed for staining intensity: 0 (not detected), 1 + (weak), 2 + (intermediate), and 3 + (strong). For each case, the histological “H-score” (0–300) was calculated by multiplying the percentage of positive cells (0–100%) by the intensity (0–3).

### Immunoblotting

A431 cells were lysed with Ripa lysis buffer (50 mM Tris-cl pH 7.4; 150 mM NaCl; 1% NP40; 0.25% Na-deoxycholate; 1 mM AEBSF; and 1 mM DTT). The total protein extracts (20 µg) were separated using SDS polyacrylamide gels and transferred on PVDF membrane, using Trans-Blot Turbo Transfer System. Then, the membranes were incubated over-night using the following antibodies: anti-SHMT2 (Sigma, Cat. No. HPA020549, 1:500), anti-CycD1 (Santa Cruz, Cat. No. sc-8396 1:500), anti-MTHFD2 (Abcam Cat. No. ab151447 1:1000), anti-p63-α (Cell Signalling, Cat. No. 13109, 1:500) and anti-vinculin (Sigma, Cat. No. V9131 1:10000). The western blot signals were captured using Alliance™ Q9-ATOM Light.

### RNA extraction and RT-qPCR

A431 cells were lysed in RLT lysis buffer (Qiagen). The Total RNA was isolated and purified using the RNeasy Mini Kit (Qiagen). The RNA was quantified using a NanoDrop spectrophotometer (Thermo Scientific). Total RNA was used for cDNA synthesis with a SensiFAST™ cDNA synthesis kit (Bioline). The qPCR was performed with a Fast SYBR™ Green Master Mix (Applied Biosystems) in an QuantStudio Real-Time PCR Systems (Applied Biosystems) using appropriate qPCR primers (Supplementary Table 1). TBP was used as housekeeping genes for data normalization. The expression of each gene was defined by the threshold cycle (Ct), and relative expression levels were calculated by using the 2 − ΔΔCt method.

### Statistical analysis

All statistical analyses were performed using GraphPad Prism 8.0 software (San Diego, CA, USA); Student’s *t* test was used for all the analysis except for confluence analysis in which One-way Anova statistical analysis was used. Values of *P* < 0.05 and *P* < 0.01 were considered statistically significant.

## Supplementary information

Supplementary Figure 1 and Supplementary Table 1 and 2

Supplementary Table 1 and 2

Original Data Files

## Data Availability

For Serine biosynthesis and One Carbon metabolism enzymes expression at mRNA level a public database available through GEO datasets (https://www.ncbi.nlm.nih.gov/gds) was analyzed (GSE7553).
