# Supplementary material for: Serine and one-carbon metabolism sustain non-melanoma skin cancer progression
Source: Cell Death Discov. 2023 Mar 24;9:102. doi: 10.1038/s41420-023-01398-x (PMC10039038; doi:10.1038/s41420-023-01398-x)
Supplement: Supplementary file 1 — Supplementary Figure 1 and Supplementary Table 1 and 2 [file 41420_2023_1398_MOESM1_ESM.pdf]

Supplementary Figure 1

Dataset GSE7553

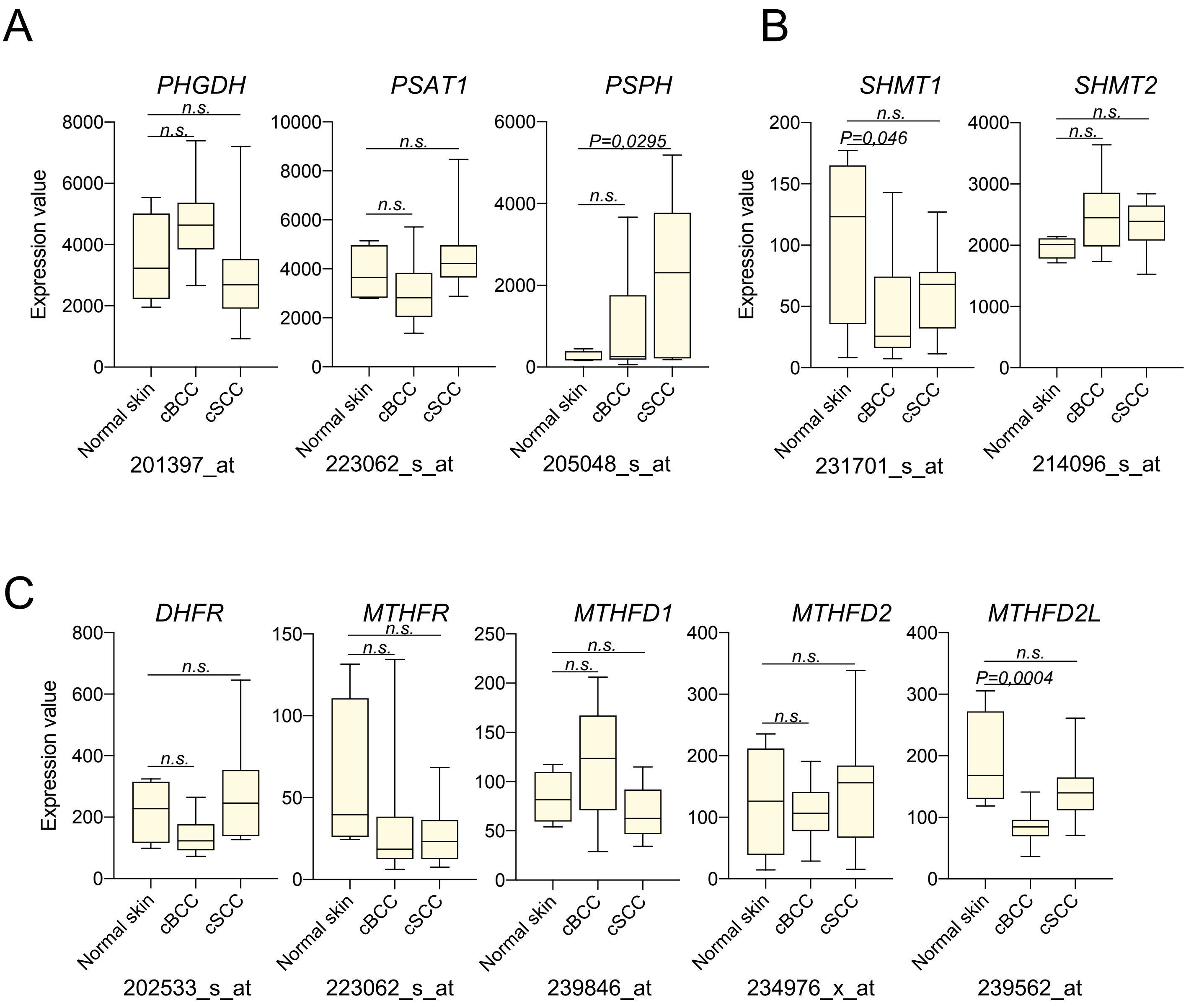

Supplementary Figure 1. Expression analysis of Serine de novo biosynthesis, Serine anabolism/catabolism and One-Carbon metabolism enzymes in a publicly available dataset of NMSC. A) Bioinformatic expression analysis performed on a publicly online dataset (GSE7553) of Serine *de novo* biosynthesis enzymes, B) *SHMT1* and *SHMT2*, involved in serine catabolism/anabolism and C) One-carbon metabolism enzymes involved in the Folate Cycle in a cohort of patients with normal skin (n=4), cBCC (n=15), cSCC (n=11). The p-value was obtained using Student's t test. Values were considered significant when the p-value<0.05, n.s.=not significant

Supplementary TABLE 1. qPCR primers

hPHGDH Fw	CACTGAGGCTGTTCCCATT
hPHGDH Rv	GTCATCAACGCAGCTGAGAA
hPSAT1 Fw	TCATCACGGACAATCACCAC
hPSAT1 Rv	GTCCTCAAACTTCCTGTCCAA
hPSPH Fw	CATGATTGGAGATGGTGCCA
hPSPH Rv	TTATCCTTGACTTGTTGCCTGA
hMTHFR Fw	GAGCGGCATGAGAGACTCC
hMTHFR Rv	CCGGTCAAACCTTGAGATGAG
hDHFR Fw	ATTTCCAGAGAATGACCACAAC
hDHFR Rv	GAGGTTCCTTGAGTTCTCTG
hMTHFD1 Fw	TTGGACAGGCTCCAACGGAGAA
hMTHFD1 Rv	AGAAGTGGTGAGAGCCAGGACA
hMTHFD2 Fw	CTACTGTGTCTTCTGTGTCAC
hMTHFD2 Rv	CTGCATGATATCGGAATGCTC
hMTHFD2L Fw	GCAATGGAATTGCCCCAGAAA
hMTHFD2L Rv	GGCAATAGGCATCCCTACGTT
hSHMT1 Fw	TGACCACCACCACCCACTCACAAGACC
hSHMT1 Rv	GCAATGGCGTGGCAACCCCAGCA
hSHMT2 Fw	AGTGATCCTGAGATGTGGGAGTT
hSHMT2 Rv	AGGATAACCCTCCGAGTACTTGTT
hTBP Fw	TCAAACCCAGAATTTGTTCTCCTTAT
hTBP Rv	CCTGAATCCCTTTAGAATAGGGTAG

Supplementary TABLE 2. siRNA sequences

hSHMT2-1	CAUUUGAGGACCGAAUCAA
hSHMT2-2	CCGGGAGAUCCCUUACACAUU
hMTHFD2-1	GGAUGCUUCACUUUGUCAA
hMTHFD2-2	UGGCAAUGCUAAUGAAGAA
