# Supplementary material for: Serine and one-carbon metabolism sustain non-melanoma skin cancer progression
Source: Cell Death Discov. 2023 Mar 24;9:102. doi: 10.1038/s41420-023-01398-x (PMC10039038; doi:10.1038/s41420-023-01398-x)
Supplement: Supplementary file 2 — Supplementary Table 1 and 2 [file 41420_2023_1398_MOESM2_ESM.docx]

Supplementary TABLE 1. qPCR primers

| hPHGDH Fw | CACTGAGGCTGTTCCCATT |
| --- | --- |
| hPHGDH Rv | GTCATCAACGCAGCTGAGAA |
| hPSAT1 Fw | TCATCACGGACAATCACCAC |
| hPSAT1 Rv | GTCCTCAAACTTCCTGTCCAA |
| hPSPH Fw | CATGATTGGAGATGGTGCCA |
| hPSPH Rv | TTATCCTTGACTTGTTGCCTGA |
| hMTHFR Fw | GAGCGGCATGAGAGACTCC |
| hMTHFR Rv | CCGGTCAAACCTTGAGATGAG |
| hDHFR Fw | ATTTCCAGAGAATGACCACAAC |
| hDHFR Rv | GAGGTTCCTTGAGTTCTCTG |
| hMTHFD1 Fw | TTGGACAGGCTCCAACGGAGAA |
| hMTHFD1 Rv | AGAAGTGGTGAGAGCCAGGACA |
| hMTHFD2 Fw | CTACTGTGTCTTCTGTGTCAC |
| hMTHFD2 Rv | CTGCATGATATCGGAATGCTC |
| hMTHFD2L Fw | GCAATGGAATTGCCCCAGAAA |
| hMTHFD2L Rv | GGCAATAGGCATCCCTACGTT |
| hSHMT1 Fw | TGACCACCACCACCCACTCACAAGACC |
| hSHMT1 Rv | GCAATGGCGTGGCAACCCCAGCA |
| hSHMT2 Fw | AGTGATCCTGAGATGTGGGAGTT |
| hSHMT2 Rv | AGGATAACCCTCCGAGTACTTGTT |
| hTBP Fw | TCAAACCCAGAATTTGTTCTCCTTAT |
| hTBP Rv | CCTGAATCCCTTTAGAATAGGGTAG |

Supplementary TABLE 2. siRNA sequences

| hSHMT2-1 | CAUUUGAGGACCGAAUCAA |
| --- | --- |
| hSHMT2-2 | CCGGGAGAUCCCUUACACAUU |
| hMTHFD2-1 | GGAUGCUUCACUUUGUCAA |
| hMTHFD2-2 | UGGCAAUGCUAAUGAAGAA |
