# Supplementary figures and images for: Serine and one-carbon metabolism sustain non-melanoma skin cancer progression
Source: Cell Death Discov. 2023 Mar 24;9:102. doi: 10.1038/s41420-023-01398-x (PMC10039038; doi:10.1038/s41420-023-01398-x)

UVITEC photo documenter

Figure 3 C

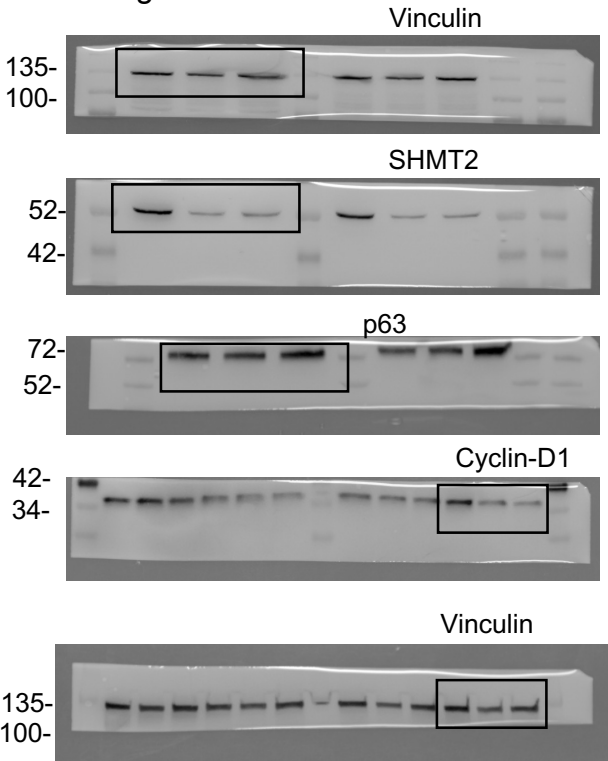

UVITEC photo documenter

Figure 3 G

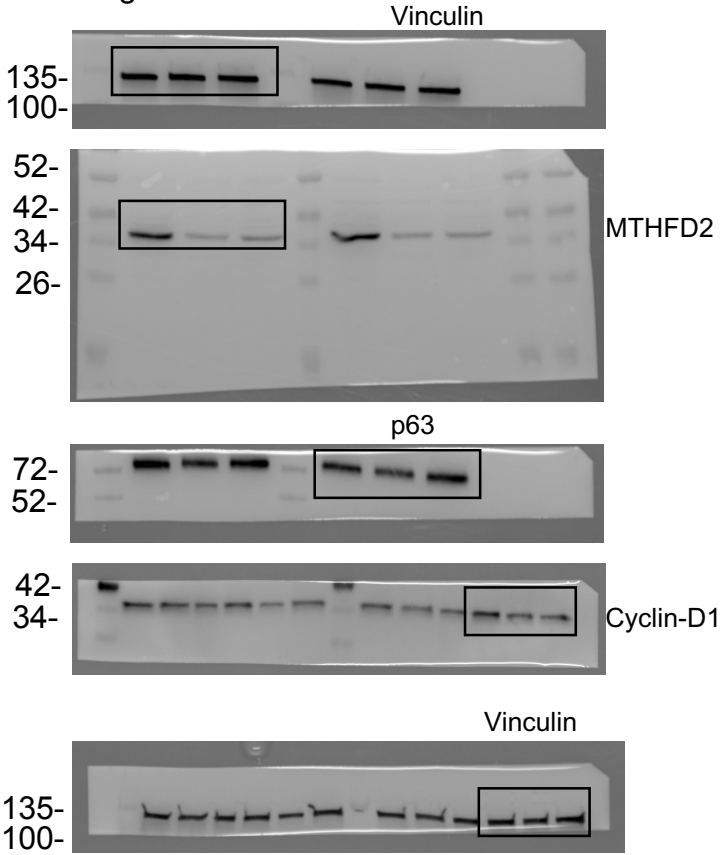

Supplement: Supplementary file 3 — Original Data Files [file 41420_2023_1398_MOESM3_ESM.pdf]
